# Blue Laser for Polymerization of Bulk-Fill Composites: Influence on Polymerization Kinetics

**DOI:** 10.3390/nano13020303

**Published:** 2023-01-11

**Authors:** Visnja Negovetic Mandic, Matej Par, Danijela Marovic, Mario Rakić, Zrinka Tarle, Eva Klarić Sever

**Affiliations:** 1 Department of Endodontics and Restorative Dentistry, School of Dental Medicine, University of Zagreb, 10000 Zagreb, Croatia; 2 Institute of Physics, 10000 Zagreb, Croatia

**Keywords:** dental materials, curing lights, dental, laser, composite resins, polymerization

## Abstract

The objective of this study was to compare the polymerization kinetics of bulk-fill resin composites cured with a LED-curing device and a diode laser (449 nm). Three bulk-fill composites were light-cured with constant radiation exposure at 10 J/cm^2^ by varying radiant exitance and curing time. The following three light-curing protocols were used: (I) 3300 mW/cm^2^ for 3 s; (II) 2000 mW/cm^2^ for 5 s; and (III) 1000 mW/cm^2^ for 10 s. The degree of conversion (DC) was monitored in real time at a data acquisition rate of 2 spectra/s over a 5-min period and again after seven days using Fourier transform infrared spectroscopy. DC amounted to 30.9–61.7% at 4-mm depth after 5 min. DC values of two sculptable composites were significantly higher with the laser, regardless of the curing protocol used, but not for the flowable composite. The maximum polymerization rate (2.0–22.1%/s) was less affected by the type of curing device for one of the composites, while the other two composites achieved significantly higher values when cured with the laser. Laser curing generally increased the DC and the maximum polymerization rate while it shortened the onset of the maximum reaction rate. New handheld laser devices with adjustable power have the potential to be used as a photopolymerization light source for new generations of bulk-fill composites.

## 1. Introduction

The use of laser technology in dentistry is on the rise again. The first use of lasers in dentistry was in 1964 for the removal of dental caries [1]. Since then, lasers of various wavelengths have become ubiquitous in almost all areas of dentistry, from hard- and soft-tissue surgery (ablation and coagulation) [2,3] to photodynamic therapy of periodontitis [4] to caries detection [5] and removal [6]. The blue laser has been successfully used for the photopolymerization of dental composite resins under experimental conditions since 1980 [7,8,9]. However, the large dimensions of the laser devices at that time and their high price discouraged dentists from using the lasers in daily practice [10]. Today, the majority of dental offices use light-emitting diode (LED)-based devices for the polymerization of composite materials [11,12].

Direct restorations with dental composite resins are the dominant treatment options for the reconstruction of destroyed tooth structure [13]. They are gaining popularity because they can mimic the natural appearance of teeth, have acceptable durability under harsh oral conditions, and can be cured with a blue light when needed [14]. With advances in the science of composite materials, the size of filler particles has been reduced to the nanoscale [15]. Nanoparticles increase the viscosity of composites and improve their polishability and hardness [16].

Light activation of the composite is possible due to its resin matrix, which consists of methacrylate monomers in the uncured, moldable state. The curing of the composite is achieved when the monomers activated by the photoinitiators enter the polymerization reaction and form a crosslinked polymer network, leading to the hardening of the material [17]. Most photoinitiators used in dental applications are activated by radiation in the blue region of the visible light spectrum. Camphorquinone, the widespread photoinitiator in most commercial products, absorbs the wavelengths in the 410–500 nm range, with an absorption maximum of 468 nm. However, due to its intense yellow color, manufacturers have moved to alternative brighter photoinitiators (phenylpropanedione, Lucirin TPO, diacylgermane-based photoinitiator Ivocerin) that are activated at lower wavelengths in the violet part of the spectrum (370–460 nm) [18]. Besides color, the alternative photoinitiator diacylgermane-based photoinitiator Ivocerin (Ivoclar Vivadent, Schaan, Liechtenstein) is one of the more efficient Norrish-type I photoinitiators, which cleave into two photoradicals at certain wavelengths [18,19]. The conventional photoinitiator, camphorquinone, is a Norrish type II sensitizer that requires a tertiary amine as a co-initiator for its photoactivation, whereupon a single radical is formed [19]. In addition, Ivocerin is more susceptible to photoactivation when irradiated at wavelengths of ~410 nm than the camphorquinone-amine system [20]. Ivocerin is also included in some dual-cure dental cements, which, in addition to photoinitiators, also contain chemical activators, mostly benzoyl peroxide (initiator) and tertiary amine (activator) as a source of free radicals [21]. Although it has been reported that certain dual-cure resin cements can be cured only with a blue light [22], photoactivation is theoretically more productive when cured with the blue and violet regions of the spectrum [18]. One of the recent “alternative” photoinitiators, Ivocerin, was developed particularly to be used in a new class of composite resins called bulk-fill composites [18].

Bulk-fill composites are the result of the trend to shorten the clinical time for composite placement by allowing placement in 4-mm layers, as opposed to 2-mm layers for conventional composites. In an effort to further reduce clinical time, a special group of bulk-fill materials is being promoted for rapid light-curing. Composite Tetric PowerFill (Ivoclar Vivadent) is the representative of this group, the composition of which features chemical modifications that enable step-like polymerization and the formation of short chains. This specific behavior is due to the addition–fragmentation chain transfer (AFCT) agent, ß-allyl sulphone, which participates in the polymerization reaction by terminating the reaction on one polymer chain and simultaneously generating another sulfone radical that allows the polymerization reaction to continue [20]. Tetric PowerFlow (Ivoclar Vivadent) is the low-viscosity bulk-fill material advertised for rapid light-curing. It does not contain a polymerization modifier, but its rapid cure is possible due to its high translucency in the unpolymerized state and low filler content. Together with Ivocerin, both materials contain camphorquinone as the main photoinitiator [20]. The rapid curing recommended by the manufacturer for the photopolymerization of these materials refers to shortening the curing time to only 3 s and increasing the irradiance of the light-curing devices to 3000 mW/cm^2^ with a violet–blue light source [20]. Ilie and Watts have confirmed that the rapid curing of Tetric PowerFill results in a similar degree of conversion (DC) to the 10 s curing at 1500 mW/cm^2^, but with faster initial polymerization kinetics than its predecessor Tetric EvoCeram Bulk Fill (Ivoclar Vivadent), which does not contain the AFCT agent [23]. However, the initially quicker polymerization kinetics and faster development of shrinkage forces [24] of Tetric PowerFill did not result in increased polymerization stress [25] and did not affect the marginal integrity on class V cavities after thermomechanical loading [26]. While the macro- and micromechanical properties of rapid curing composites were satisfactory in the upper 2 mm [23,27], a significantly lower flexural modulus was observed for the depth of 2–4 mm [28].

Therefore, the curing equipment used for the photoactivation of rapid-curing composites had to be adapted to these changes. Nowadays, LED-curing devices with irradiance above 3000 mW/cm^2^ and violet–blue bandwidth are available from several manufacturers. LEDs are generally considered durable and are known for their long lifetime [10]. However, their irradiance decreases with time, similar to older quartz–tungsten–halogen devices, although at a much slower rate. The emission of higher optical power from LED chips leads to an increase in their temperature. It is well known that thermal stress leads to the degradation of the optical power of LEDs, which eventually shortens their lifetime [29]. At the same time, laser devices are becoming smaller, battery-powered, and have an adjustable spot size [30,31,32]. The narrow emission spectrum of laser light enables precise targeting to the maximum absorption spectrum of photoinitiators. This feature has contributed to the higher DC in previous studies [7,8,9]. Having overcome the disadvantages of laser light in the past, they have now reemerged as a possible alternative to high-power LED-curing devices.

The aim of this study was to evaluate the use of a hand-held diode laser with adjustable irradiance for the polymerization of a new generation of rapid-curing bulk-fill composites. The following parameters were tested: polymerization kinetic parameters, maximum polymerization rate, time to reach maximum polymerization rate, and DC after 5 min and after seven days. The following null hypotheses were made:

(I) there is no difference between the laser light and commercial LED-light-curing device in the tested parameters;
(II) there is no difference between different curing protocols with the same radiant exposure;
(III) there is no difference in polymerization kinetics among materials.

## 2. Materials and Methods

**Figure 1:**
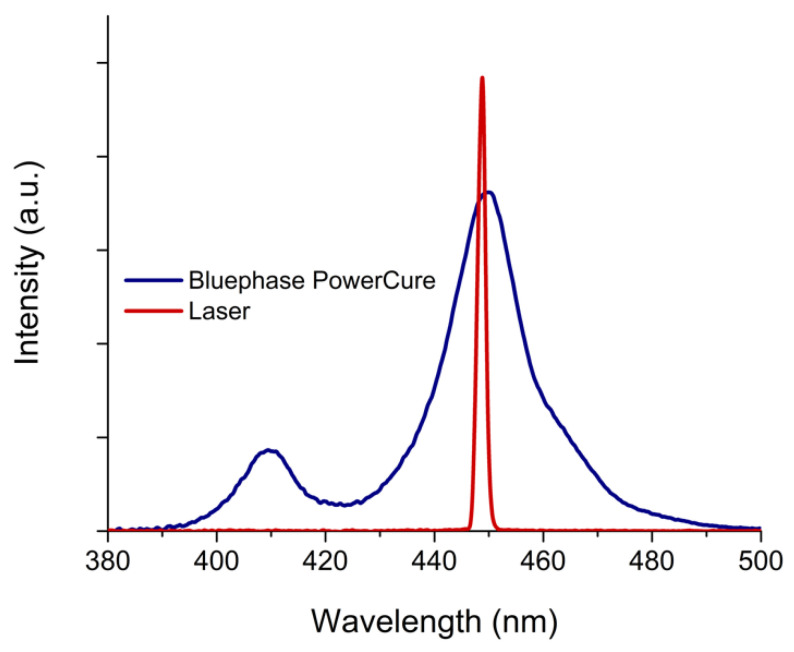
Emission spectrum of the laser and Bluephase PowerCure used in this study. Intensity of the laser spectrum is scaled down for better visualization.

Three contemporary bulk-fill composites were used in this study (Table 1). Composite specimens (*n* = 8) were prepared in custom-made silicone molds (diameter = 3 mm, height = 4 mm) on top of a diamond-attenuated total-reflectance (ATR) crystal and light-cured using either a diode laser (449 nm, 1.6 W, Jinjiang Co., Quanzhou City, China) or a conventional LED-curing unit (Bluephase PowerCure, Ivoclar Vivadent, Schaan, Liechtenstein). The radiant exitance and the spectral emission of both laser and the Bluephase PowerCure were measured with a calibrated and NIST-referenced UV-Vis spectrophotometer system (MARC; BlueLight Analytics, Halifax, Canada) and shown in Figure 1.

The following three light-curing protocols were used, keeping the radiant exposure at 10 J/cm^2^:

(I) 3300 mW/cm^2^ for 3 s;
(II) 2000 mW/cm^2^ for 5 s;
(III) 1000 mW/cm^2^ for 10 s.

### 2.1. Polymerization Kinetics

Polymerization kinetics was evaluated using the Fourier transform infrared (FTIR) spectrometer (Nicolet iS50, Thermo Fisher, Madison, WI, USA) with an attenuated total reflectance (ATR) accessory. The FTIR spectra were acquired in real time at a rate of 2 spectra/s for 5 min, with four scans per spectrum and a spectral resolution of 8 cm^−1^ [33].

The changes in the ratio of absorbance intensities of the aliphatic band at 1638 cm^−1^ and aromatic band at 1608 cm^−1^ were used to calculate DC:

(1) $$DC\;\left(\%\right)=\left[1-\frac{{\left(1638\;cm^{-1}/1608\;cm^{-1}\right)}_{peak\;height\;after\;curing}\;}{{\left(1638\;cm^{-1}/1608\;cm^{-1}\right)}_{peak\;height\;before\;curing}}\right]\times 100$$

**Figure 2:**
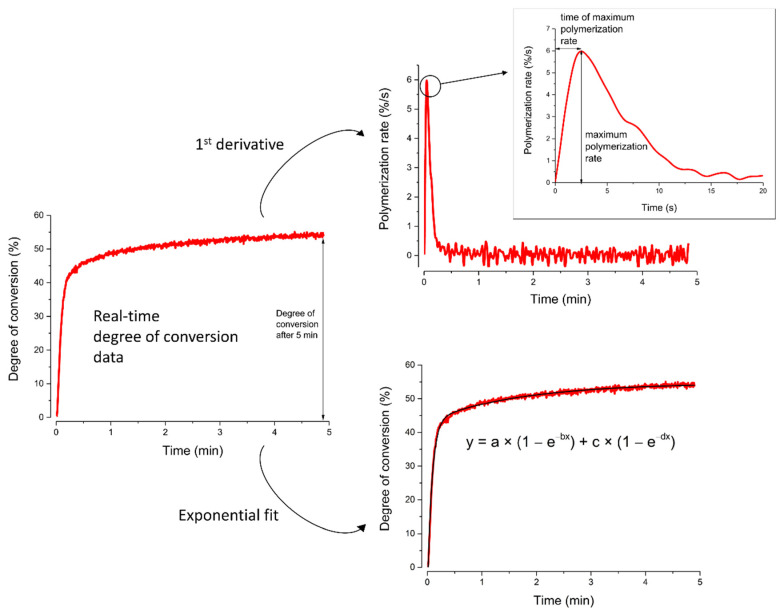
Illustration of the experimental method.

The DC data were plotted as a function of time, and the first derivatives were calculated to represent the reaction rate (Figure 2). The obtained reaction rate was plotted as a function of time to determine the maximum reaction rate (R_max_) and time to reach the maximum reaction rate (t_max_). Additionally, the DC values achieved at the end of the 5-min observation period (DC_5 min_) were evaluated.

A four-parameter exponential sum function fitted the curves of DC against time.

y = a × (1 − e^−bx^) + c × (1 − e^−dx^) (2)

The four modulation parameters in this equation are used to describe the polymerization kinetics during the gel phase (parameters a and b) and the glass phase (parameters c and d).

### 2.2. Post-Cure DC Evaluation

The specimens (n = 8) used for the evaluation of polymerization kinetics were dark-stored at 37 °C for seven days. The aforementioned ATR-FTIR instrument was used to determine the DC. The top specimen surface, i.e., the surface that faced the curing unit during the illumination, was pressed against the diamond ATR crystal, and 30 FTIR spectra were recorded (4 scans per spectrum) at a spectral resolution of 8 cm^−1^. DC was calculated according to Equation (1).

### 2.3. Statistical Analysis

After verifying the normality of distribution using Shapiro–Wilk’s test, data were compared using parametric statistics. DC after 5 min, DC after seven days, maximum polymerization rate, and time to reach the maximum polymerization rate were compared considering “material”, “curing unit”, and “curing protocol” factors and using a three-way ANOVA. Since statistically significant interactions of these three factors were observed, further statistical analysis was performed using two separate one-way ANOVAs to identify the effects of the “material” (3 levels) and “curing protocol” (3 levels) factors, while an independent-observation *t*-test was used for comparisons between curing units (2 levels). Tukey’s post-hoc adjustment was used for multiple comparisons. Statistical analysis was performed using SPSS (version 25; IBM, Armonk, NY, USA) at an overall significance level of 0.05.

## 3. Results

### 3.1. Polymerization Kinetics

**Figure 3:**
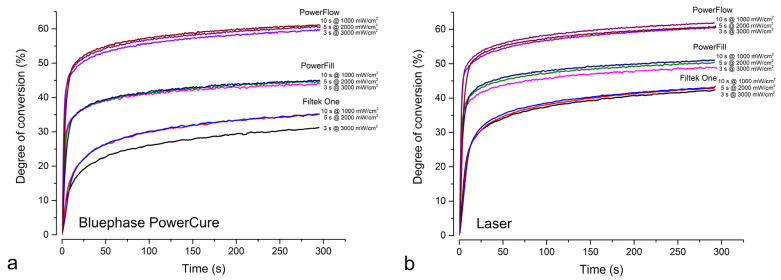
Averaged curves (n = 8) of degree of conversion at the bottom of 4-mm layers as a function of time. (**a**) Bluephase PowerCure; (**b**) Laser.

Figure 3 shows the evolution of DC in real time during 5 min immediately after illumination. Different behavior was observed for the different materials, while the polymerization protocols did not show much difference, except for the 3 s curing of Filtek One. In both laser and Bluephase PowerCure polymerization, the flowable material Tetric PowerFlow reached the highest DC value within the first 10 s in all polymerization protocols, followed by Tetric PowerFill and Filtek One.

**Figure 4:**
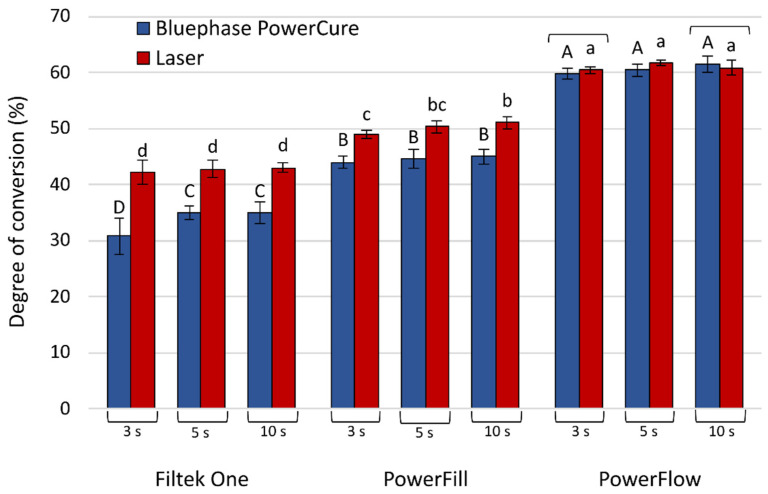
Degree of conversion (mean values ± standard deviations) measured after 5 min. Statistically homogeneous groups are indicated by same uppercase letters for Bluephase PowerCure and same lowercase letters for the laser. Square brackets indicate statistically similar values for comparisons of Bluephase PowerCure vs. laser.

Figure 4 shows statistical comparisons of DC values reached 5 min post-cure between laser and Bluephase PowerCure. Laser curing showed higher DC than Bluephase PowerCure in all cases, except for the Tetric PowerFlow at 3 s and 10 s polymerization, where no statistically significant difference was found between the light-curing unit and the laser.

**Figure 5:**
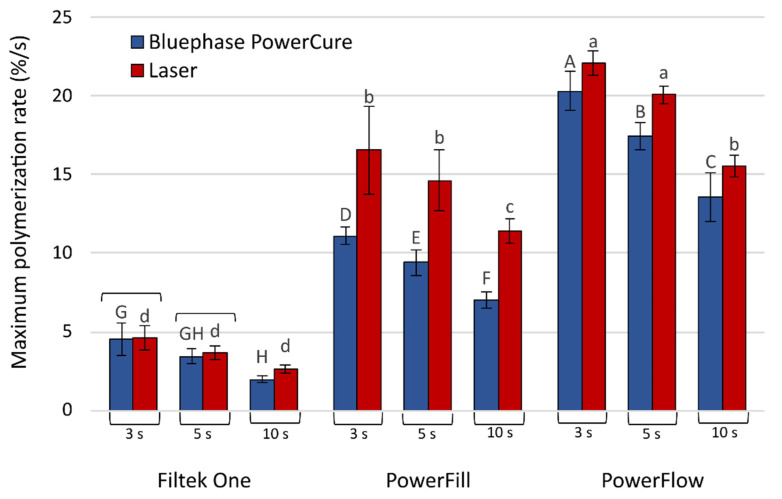
Maximum polymerization reaction rate (mean values ± standard deviations). Statistically homogeneous groups are indicated by same uppercase letters for Bluephase PowerCure and same lowercase letters for the laser. Square brackets indicate statistically similar values for comparisons of Bluephase PowerCure vs. laser.

The maximum polymerization rate (Figure 5) was the slowest for Filtek One at 10 s polymerization with Bluephase PowerCure. For the same material, there was no difference between laser and Bluephase PowerCure at 3 s and 5 s polymerization. For all other materials and curing protocols, laser curing resulted in faster polymerization rates, the fastest being the 3 s polymerization.

**Figure 6:**
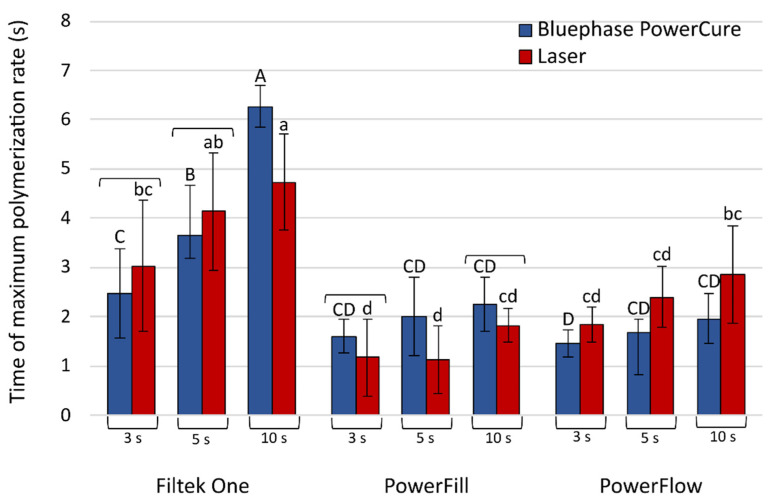
Time to achieve maximum polymerization rate (mean values ± standard deviations). Statistically homogeneous groups are indicated by same uppercase letters for Bluephase PowerCure and same lowercase letters for the laser. Square brackets indicate statistically similar values for comparisons of Bluephase PowerCure vs. laser.

Conversely, the time to reach the maximum polymerization rate (Figure 6) was the longest for Filtek One, especially for 10 s polymerization with Bluephase PowerCure. The time to reach the maximum polymerization rate for 10 s laser irradiation of the same material was considerably shorter. Tetric PowerFill and Tetric PowerFlow reached their maximum polymerization rate in 1.1–2.9 s.

The polymerization kinetics parameters a, b, c, and d denote the gel phase (a and b) and the glass phase (c and d) of the polymerization process [33]. Table 2 shows that the highest values of the “a” parameter were achieved with the Tetric PowerFlow and were higher for the laser than for the Bluephase PowerCure, with values increasing slightly with increasing polymerization time. On the other hand, curing with Bluephase PowerCure for Filtek One had the lowest “a” parameter at 3 s, which increased at 5 s and 10 s curing. The opposite was true for parameter “c”, which was the highest for Filtek One and the lowest for Tetric PowerFill and Tetric PowerFlow. The values of parameters “b” and “d” were low for all materials and polymerizations.

### 3.2. Post-Cure DC Development

**Figure 7:**
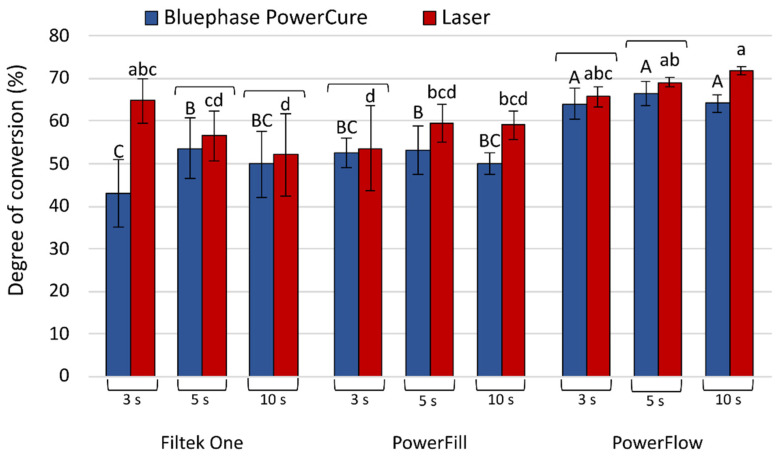
Degree of conversion values measured at top specimen surfaces 7 days post-cure (mean values ± standard deviations). Statistically homogeneous groups are indicated by same uppercase letters for Bluephase PowerCure and same lowercase letters for the laser. Square brackets indicate statistically similar values for comparisons of Bluephase PowerCure vs. laser.

The DC measured on the top sides seven days after curing is shown in Figure 7. The post-cure increase of DC was observed in all tested materials. The highest increase, which was 22.2%, was measured for Filtek One, which was polymerized for 3 s with the laser. The post-cure evolution DC balanced the differences between the laser and Bluephase PowerCure. After seven days, laser and Bluephase PowerCure performed equally well for Filtek One at 5 s and 10 s curing, for Tetric PowerFill at 3 s and 5 s curing, and for Tetric PowerFlow at 3 s and 5 s curing.

## 4. Discussion

In this study, the effect of photopolymerization of currently commercially available bulk-fill composites was investigated using a laser or a conventional LED-light-curing device. Two of the three materials tested here were specifically designed for rapid curing with a high-light intensity device. Our results showed a material-dependent behavior with higher DC values for laser curing for high-viscosity materials and equal DC values for low-viscosity materials. For most groups studied, laser curing accelerated the polymerization reaction, resulting in a higher maximum polymerization rate and a reduction in the time to reach the maximum polymerization rate. The well-known increase in DC [34] after curing was also observed in this study after seven days, with the highest increase in the materials that had the lowest DC in the 5-min measurement. Since statistically significant effects of the curing unit type, curing protocol, and material were observed, all three null hypotheses were rejected.

For this study, three bulk materials with different compositions were selected. The common feature of all tested materials is the change in the refractive index of the resin during light curing, leading to an increased opacity at the end of illumination, which is desirable for esthetic purposes [20,35]. Although both Tetric materials, PowerFill and PowerFlow, are advertised as the materials for fast curing, only Tetric PowerFill contains the AFCT agent, ß-allyl sulphone. According to the manufacturer, both Tetric PowerFlow and Tetric PowerFill initially exhibit well-matched refractive indices of the unpolymerized resin matrix and the fillers, which no longer match after resin polymerization, resulting in increased light attenuation and opacity of the composite [20]. This behavior is likely more pronounced with Tetric PowerFlow due to the comparatively lower filler volume [36]. Filtek One is the material with the highest filler volume of the three materials tested. Its distinctive feature is the high molecular weight monomer aromatic urethane dimethacrylate (AUDMA), which was added to reduce polymerization shrinkage. The manufacturer also claims that Filtek One contains an addition–fragmentation monomer (AFM). However, unlike the AFCT agent in Tetric PowerFill, the AFM monomer in Filtek One is only “activated” when the material is subjected to high polymerization stress. In this case, the AFM fragments the polymeric network and reattaches after the stress has been relieved [35].

Thus, despite their apparent similarity, the two high-viscosity bulk-fill materials tested are fundamentally different, which is confirmed by our results. Rapid curing at high intensity, either by LED or by the laser device, is not sufficient for the effective polymerization of Filtek One in the early stage of polymerization (5 min). The initial high viscosity due to the high filler volume and the low mobility of the long chain AUDMA are the likely explanations for the lowest DC values at the 5-min time point. AUDMA has only two methacrylate groups, and the long-chain molecule has limited mobility, making it difficult to bring the methacrylate groups into close physical proximity. Both high filler volume and the long-chain AUDMA restrict the mobility of the radical species and hinder the interaction with monomers to form the polymer chains [16]. This assumption is confirmed by the lowest maximum polymerization rate of Filtek One, regardless of the curing device (Bluephase PowerCure or laser). In terms of the illumination duration, short curing times (3 s with 3300 mW/cm^2^ and 5 s with 2000 mW/cm^2^) resulted in a similarly low maximum polymerization rate, but the time to reach this rate was longer with the longer curing times. In this study, the longer curing time corresponded to the lower light exitance of the curing device to achieve the same radiant exposure of 10 J/cm^2^. A longer curing time with a lower light output was obviously more beneficial for the polymerization of Filtek One since a higher parameter “a” and DC were observed at the 5-min curing time. A similar conclusion was drawn in our previous studies with the same material [28,37].

The laser beam, on the other hand, is less divergent than the light from LED because of its coherent, collimated light beam [38,39]. When laser light passes through the material, some of the photons are absorbed, but it does not lose intensity as the light from the light-curing device due to the dense, parallel photon beam. Therefore, the laser has a lower light attenuation [38,40] than the LED light used here and effectively activates the photoinitiators for both high-viscosity materials regardless of curing time. As a highly translucent material, the flowable composite Tetric PowerFlow allows uniform transmission for both the laser and LED light. In contrast to the LED-curing unit, there was no difference in the 5-min DC values between different curing times with the laser. At this point, it is important to emphasize that the laser power was also set to 10 J/cm^2^ radiant exposure to ensure the same experimental conditions. Therefore, greater differences were found among various materials in the final 5-min or seven-day DC values than between the laser curing times. Similarly, the differences between the polymerization kinetic parameters “a” and “c” between 3, 5, or 10 s laser curing were negligible. The only differences between the different times of laser curing were observed in the maximum reaction rate, which became slower as the curing time increased. This behavior is most likely related to the lower radiation power associated with longer curing time. The time to reach the maximum polymerization rate also increased with the increasing duration of laser curing, with the exception of Tetric PowerFill, where the time to reach the maximum polymerization rate was the same for each duration of laser curing.

The decrease in polymerization rate and the increase in time to reach the maximum polymerization rate were notable with increasing curing time in both laser and LED-light curing. This behavior can be easily explained by the lower radiant exitance that accompanied a longer curing time. For the 10-s group, a smaller number of released photons passed through the 4-mm-thick sample for a longer period of time, resulting in a smaller number of photoinitiator molecules being activated than for the 3-s group. The activated photoinitiators produced a smaller number of initiation spots, which slowed down the polymerization reaction and prolonged the time to reach the maximum polymerization rate. An exception was Tetric PowerFill, which contains the polymerization modulator ß-allyl sulphone, which doubles the number of free radicals and the number of initiation spots. In addition, the germanium-based photoinitiator Ivocerin belongs to the Norrish-type photoinitiators II, which means that one photoinitiator molecule generates two free radicals. Both factors are likely to have contributed to the fact that the time to reach the maximum polymerization rate in Tetric PowerFill was shortened.

A surprising finding of this study was a generally higher 5-min DC for Ivocerin-containing material Tetric PowerFill with blue-laser curing than with the violet–blue LED device. The maximum absorption wavelengths for Ivocerin range from 370–460 nm, with a peak sensitivity of around 410 nm. A diode laser with a narrow emission wavelength centered around 449 nm is, therefore, on the outer margin for activation of Ivocerin. However, Tetric PowerFill also contains camphorquinone, which, with its absorption maximum at 468 nm, perfectly matches the emission spectra of the investigated diode laser. This suggests that the deep penetrability of the laser’s coherent monochromatic blue radiation is able to activate the alternative photoinitiator, Ivocerin, as well as camphorquinone. The photon beam emitted by the laser source apparently activates more photoinitiator molecules in the 4-mm layer than the LED-curing device under the same radiant exposure [41]. In addition, it is known that a shorter violet light has a lower penetrating power than blue light and is more scattered, which leads to the activation of photoinitiator molecules in the superficial layers [42]. Since we measured polymerization kinetics at the 4-mm level, it is likely that we did not observe the effect of the additional photoinitiator that might be present at the sample surface. However, DC data measured after seven days on the specimen top surfaces also indicated that laser curing led to higher or statistically similar values compared to the violet–blue LED-curing unit. Similarly, higher 5-min DC values were also observed for Filtek One when it was cured with a laser rather than a curing device. Hence, it appears that using the narrow range of laser light was beneficial for the curing of both tested high-viscosity bulk-fill composites regardless of the presence of alternative violet-light-absorbing photoinitiators. An analogous effect of DC improvement produced by laser curing was also identified for the flowable composite (PowerFlow); however, it had a lower effect size, and the corresponding statistical significance was observed only in some experimental groups (5 s curing for 5-min DC measurements and 10 s curing for seven-day measurements).

## 5. Conclusions

The new laser devices are hand-held devices with adjustable power levels that can be used as a light source for photopolymerization in new generations of bulk-fill composites. The laser device used here generally resulted in a higher or equal degree of conversion compared to a reference LED-light-curing device with cure times as short as 3 s. The laser light accelerated the polymerization reaction, which would likely translate into a clinically undesirable increase in polymerization shrinkage and stress; hence further studies of these topics are granted.

## Figures and Tables

**Table 1:** Composition of tested materials provided by the manufacturers.

Material	Manufacturer	Resin	Filler wt%/vol%	Photoinitiator
Filtek One Bulk Fill Restorative	3M ESPE, St. Paul, MN, USA	AUDMA, DDDMA, proprietary AFM	76.5/58.5	CQ/amine
Tetric PowerFill	Ivoclar Vivadent, Schaan, Liechtenstein	Bis-GMA, Bis-EMA, UDMA, PBPA, DCP, β-allyl sulfone	76/53	CQ/amine Ivocerin
Tetric PowerFlow	Ivoclar Vivadent, Schaan, Liechtenstein	Bis-GMA, Bis-EMA, UDMA, DCP	68/46	CQ/amine Ivocerin

Bis-GMA: bisphenol A-diglycidyl dimethacrylate, UDMA: urethane dimethacrylate; Bis-EMA: ethoxylated bisphenol A dimethacrylate; DCP: tricyclodecane-dimethanol dimethacrylate; PBPA: propoxylated bisphenol A dimethacrylate; CQ: camphorquinone; AUDMA: aromatic urethane dimethacrylate; DDDMA: 1, 12-dodecanediol dimethacrylate; AFM: addition-fragmentation monomer; TEGDMA: triethylene glycol dimethacrylate.

**Table 2:** Fit parameters a, b, c, and d of exponential function (limits of 95% confidence interval in parentheses).

		Bluephase PowerCure			Laser		
		Filtek One	PowerFill	PowerFlow	Filtek One	PowerFill	PowerFlow
a	**3 s @ 3000 mW/cm^2^**	15.63 (15.11–16.15)	35.90 (34.77–37.03)	49.28 (47.73–50.82)	30.37 (29.81–30.93)	40.96 (39.80–42.12)	54.53 (52.89–56.18)
	**5 s @ 2000 mW/cm^2^**	19.87 (19.34–20.41)	36.87 (35.90–37.85)	51.87 (50.55–53.20)	32.35 (31.64–33.07)	41.74 (40.77–42.71)	54.18 (52.93–55.43)
	**10 s @ 1000 mW/cm^2^**	22.38 (21.92–22.85)	36.89 (36.24–37.54)	52.69 (51.77–53.60)	33.89 (33.26–34.53)	44.14 (43.37–44.91)	56.86 (55.81–57.90)
b	**3 s @ 3000 mW/cm^2^**	0.12 (0.11–0.13)	0.43 (0.41–0.46)	0.51 (0.48–0.54)	0.16 (0.15–0.16)	0.49 (0.47–0.51)	0.51 (0.48–0.54)
	**5 s @ 2000 mW/cm^2^**	0.13 (0.12–0.13)	0.32 (0.31–0.34)	0.41 (0.39–0.43)	0.14 (0.13–0.15)	0.44 (0.42–0.46)	0.46 (0.44–0.48)
	**10 s @ 1000 mW/cm^2^**	0.11 (0.10–0.11)	0.23 (0.22–0.24)	0.29 (0.28–0.29)	0.12 (0.12–0.13)	0.28 (0.27–0.29)	0.24 (0.24–0.25)
c	**3 s @ 3000 mW/cm^2^**	14.32 (14.06–14.57)	10.05 (9.75–10.35)	12.72 (12.36–13.08)	14.38 (14.14–14.61)	10.10 (9.83–10.36)	11.36 (11.01–11.71)
	**5 s @ 2000 mW/cm^2^**	15.45 (15.19–15.71)	10.47 (10.20–10.75)	12.26 (11.93–12.60)	14.52 (14.20–14.84)	10.05 (9.81–10.28)	11.28 (10.99–11.57)
	**10 s @ 1000 mW/cm^2^**	14.68 (14.42–14.94)	10.18 (9.96–10.40)	11.65 (11.38–11.93)	13.12 (12.82–13.42)	9.54 (9.30–9.77)	9.99 (9.67–10.31)
d	**3 s @ 3000 mW/cm^2^**	0.01 (0.01–0.01)	0.01 (0.01–0.01)	0.01 (0.01–0.01)	0.01 (0.01–0.01)	0.01 (0.01–0.01)	0.01 (0.01–0.01)
	**5 s @ 2000 mW/cm^2^**	0.01 (0.01–0.01)	0.01 (0.01–0.01)	0.01 (0.01–0.01)	0.01 (0.01–0.01)	0.01 (0.01–0.01)	0.01 (0.01–0.01)
	**10 s @ 1000 mW/cm^2^**	0.01 (0.01–0.01)	0.01 (0.01–0.01)	0.01 (0.01–0.01)	0.01 (0.01–0.01)	0.01 (0.01–0.01)	0.01 (0.01–0.01)

## Data Availability

Data are available from corresponding authors on reasonable request.

## References

[1] Goldman L., Hornby P., Meyer R., Goldman B. (1964). Impact of the Laser on Dental Caries. Nature.

[2] Pick R.M., Pecaro B.C. (1987). Use of the CO_2_ laser in soft tissue dental surgery. Lasers Surg. Med..

[3] Strauss R.A., Fallon S.D. (2004). Lasers in contemporary oral and maxillofacial surgery. Dent. Clin. N. Am..

[4] Salvi G.E., Stahli A., Schmidt J.C., Ramseier C.A., Sculean A., Walter C. (2020). Adjunctive laser or antimicrobial photodynamic therapy to non-surgical mechanical instrumentation in patients with untreated periodontitis: A systematic review and meta-analysis. J. Clin. Periodontol..

[5] Matosevic D., Tarle Z., Miljanić S., Meic Z., Pichler L., Pichler G. (2010). Laser induced Fluorescence of Carious lesion Porphyrins. Acta Stomatol. Croat..

[6] Montedori A., Abraha I., Orso M., D’Errico P.G., Pagano S., Lombardo G. (2016). Lasers for caries removal in deciduous and permanent teeth. Cochrane Database Syst. Rev..

[7] Tarle Z., Meniga A., Ristic M., Sutalo J., Pichler G. (1995). Polymerization of composites using pulsed laser. Eur. J. Oral Sci..

[8] Meniga A., Tarle Z., Ristic M., Sutalo J., Pichler G. (1997). Pulsed blue laser curing of hybrid composite resins. Biomaterials.

[9] Severin C., Macchi-Grisot F. (1980). Polymerization of Bowen’s resins by ultraviolet laser beam. Ligament.

[10] Santini A. (2010). Current status of visible light activation units and the curing of light-activated resin-based composite materials. Dent. Update.

[11] Dederich D.N., Bushick R.D. (2004). Lasers in dentistry: Separating science from hype. J. Am. Dent. Assoc..

[12] Matošević D., Pandurić V., Janković B., Knežević A., Klarić E., Tarle Z. (2011). Light Intensity of Curing Units in Dental Offices in Zagreb, Croatia. Acta Stomatol. Croat..

[13] Ferracane J.L. (2011). Resin composite—State of the art. Dent. Mater..

[14] Haugen H.J., Marovic D., Par M., Thieu M.K.L., Reseland J.E., Johnsen G.F. (2020). Bulk Fill Composites Have Similar Performance to Conventional Dental Composites. Int. J. Mol. Sci..

[15] Ilie N., Hickel R. (2009). Macro-, micro- and nano-mechanical investigations on silorane and methacrylate-based composites. Dent. Mater..

[16] Halvorson R.H., Erickson R.L., Davidson C.L. (2003). The effect of filler and silane content on conversion of resin-based composite. Dent. Mater..

[17] Marovic D., Panduric V., Tarle Z., Ristic M., Sariri K., Demoli N., Klaric E., Jankovic B., Prskalo K. (2013). Degree of conversion and microhardness of dental composite resin materials. J. Mol. Struct..

[18] Moszner N., Fischer U.K., Ganster B., Liska R., Rheinberger V. (2008). Benzoyl germanium derivatives as novel visible light photoinitiators for dental materials. Dent. Mater..

[19] Ilie N. (2022). Resin-Based Bulk-Fill Composites: Tried and Tested, New Trends, and Evaluation Compared to Human Dentin. Materials.

[20] Todd J. (2019). Scientific Documentation 3s PowerCure.

[21] Carek A., Dukaric K., Miler H., Marovic D., Tarle Z., Par M. (2022). Post-Cure Development of the Degree of Conversion and Mechanical Properties of Dual-Curing Resin Cements. Polymers.

[22] Braga S.S.L., Price R.B., Juckes S.M., Sullivan B., Soares C.J. (2022). Effect of the violet light from polywave light-polymerizing units on two resin cements that use different photoinitiators. J. Prosthet. Dent..

[23] Ilie N., Watts D.C. (2020). Outcomes of ultra-fast (3 s) photo-cure in a RAFT-modified resin-composite. Dent. Mater..

[24] Par M., Marovic D., Attin T., Tarle Z., Taubock T.T. (2020). Effect of rapid high-intensity light-curing on polymerization shrinkage properties of conventional and bulk-fill composites. J. Dent..

[25] Par M., Burrer P., Prskalo K., Schmid S., Schubiger A.L., Marovic D., Tarle Z., Attin T., Taubock T.T. (2022). Polymerization Kinetics and Development of Polymerization Shrinkage Stress in Rapid High-Intensity Light-Curing. Polymers.

[26] Par M., Spanovic N., Marovic D., Attin T., Tarle Z., Taubock T.T. (2021). Rapid high-intensity light-curing of bulk-fill composites: A quantitative analysis of marginal integrity. J. Dent..

[27] Par M., Marovic D., Attin T., Tarle Z., Taubock T.T. (2020). The effect of rapid high-intensity light-curing on micromechanical properties of bulk-fill and conventional resin composites. Sci. Rep..

[28] Marovic D., Par M., Crnadak A., Sekelja A., Negovetic Mandic V., Gamulin O., Rakic M., Tarle Z. (2021). Rapid 3 s Curing: What Happens in Deep Layers of New Bulk-Fill Composites?. Materials.

[29] Trevisanello L., Meneghini M., Mura G., Sanna C., Buso S., Spiazzi G., Vanzi M., Meneghesso G., Zanoni E. (2007). Thermal stability analysis of High Brightness LED during high temperature and electrical aging. Proc. SPIE-Int. Soc. Opt. Eng..

[30] Li C., Liu P., Shao P., Pei J., Li Y., Pawlik T.M., Martin E.W., Xu R.X. (2019). Handheld projective imaging device for near-infrared fluorescence imaging and intraoperative guidance of sentinel lymph node resection. J. Biomed. Opt..

[31] Arrizabalaga-Larrañaga A., Nielen M.W.F., Blokland M.H. (2021). Hand-Held Diode Laser for On-Site Analysis Using Transportable Mass Spectrometry. Anal. Chem..

[32] Wheeland R.G. (2007). Simulated consumer use of a battery-powered, hand-held, portable diode laser (810 nm) for hair removal: A safety, efficacy and ease-of-use study. Lasers Surg. Med..

[33] Ilie N. (2017). Impact of light transmittance mode on polymerisation kinetics in bulk-fill resin-based composites. J. Dent..

[34] Par M., Lapas-Barisic M., Gamulin O., Panduric V., Spanovic N., Tarle Z. (2016). Long Term Degree of Conversion of two Bulk-Fill Composites. Acta Stomatol. Croat..

[35] (2020). 3M. Filtek One Bulk Fill Restaurative. Shrinkage, Stress and Bulk Fill Restoratives. https://multimedia.3m.com/mws/media/1317665O/1317663m-filtek-one-bulk-fill-restorative-shrink-stressand-bulk-fill-restoratives.pdf.

[36] Arikawa H., Kanie T., Fujii K., Takahashi H., Ban S. (2007). Effect of filler properties in composite resins on light transmittance characteristics and color. Dent. Mater. J..

[37] Klarić N., Macan M., Par M., Tarle Z., Marović D. (2022). Effect of Rapid Polymerization on Water Sorption and Solubility of Bulk-fill Composites. Acta Stomatol. Croat..

[38] Delfino C., Pfeifer C., Braga R., Youssef M., Turbino M. (2009). Shrinkage stress and mechanical properties of photoactivated composite resin using the argon ion laser. Appl. Phys. B.

[39] Svelto O. (2010). Principles of Lasers.

[40] Fleming M.G., Maillet W.A. (1999). Photopolymerization of composite resin using the argon laser. J. Can. Dent. Assoc..

[41] Rocha M.G., Maucoski C., Roulet J.F., Price R.B. (2022). Depth of cure of 10 resin-based composites light-activated using a laser diode, multi-peak, and single-peak light-emitting diode curing lights. J. Dent..

[42] Harlow J.E., Rueggeberg F.A., Labrie D., Sullivan B., Price R.B. (2016). Transmission of violet and blue light through conventional (layered) and bulk cured resin-based composites. J. Dent..

